# Mapping the inclusion of affirmative policies in postgraduate nursing courses

**DOI:** 10.1590/1980-220X-REEUSP-2023-0087en

**Published:** 2023-09-22

**Authors:** Jhennifer Nycole Rocha da Silva de Castro, Nyvia Cristina dos Santos Lima, Iago Sérgio de Castro Farias, Deisiane da Silva Mesquita, Karytta Sousa Naka, Ingrid Fabiane Santos da Silva, Andressa Tavares Parente, Nádile Juliane Costa de Castro

**Affiliations:** 1 Universidade Federal do Pará, Faculdade de Enfermagem, Belém, PA, Brazil.; 2 Universidade Federal do Pará, Programa de Pós-graduação em Enfermagem, Belém, PA, Brazil.; 3 Fundação Oswaldo Cruz, Programa de Pós-graduação em Saúde Pública e Meio Ambiente, Rio de Janeiro, RJ, Brazil.; 4 Faculdade Estácio, Castanhal, PA, Brazil.; 5 Universidade Federal do Rio de Janeiro, Escola de Enfermagem Anna Nery, Rio de Janeiro, RJ, Brazil.

**Keywords:** Public Policy, Social Vulnerability, Education, Nursing, Graduate, Teaching, Social Inclusion, Política Pública, Vulnerabilidad Social, Educación de Postgrado en Enfermería, Enseñanza, Inclusión Social, Política Pública, Vulnerabilidade Social, Educação de Pós-Graduação em Enfermagem, Ensino, Inclusão social

## Abstract

**Objective:**

To map the inclusion of affirmative policies in Postgraduate Nursing courses in Brazil.

**Method:**

This is a descriptive, document-based study, carried out with information collected on the Sucupira Platform, via the Coordination for the Improvement of Higher Education Personnel, linked to the Ministry of Education. After data collection, carried out between October 2021 and March 2022, each public notice was read in full, in order to extract the following elements: name of the program; program code; name of the Higher Education Institution; acronym; Federative unit; and number of vacancies for master’s courses.

**Results:**

79 institutions in the country were identified, with a final sample of 67 evaluated programs, which were classified into nine types, based on the use of affirmative policy principles in academic and professional master’s degrees.

**Conclusion:**

Postgraduate Nursing courses present an imbalance regarding the implementation of affirmative policies in their offers of regular vacancies, as well as in issues of equity in regional access and diversity in the social groups contemplated.

## RESUMO

**Objetivo:**

Mapear a inclusão de políticas afirmativas nos cursos de pós-graduação em Enfermagem no Brasil.

**Método:**

Trata-se de um estudo descritivo e de base documental, realizado com informações coletadas na Plataforma Sucupira, via Coordenação de Aperfeiçoamento de Pessoal de Nível Superior que é vinculada ao Ministério da Educação. Após a coleta de dados, feita entre outubro de 2021 e março de 2022, deu-se a leitura integral de cada edital para a extração dos seguintes elementos: nome do programa; código do programa; nome da Instituição de Ensino Superior; sigla; Unidade Federativa; quantitativos de vagas para cursos de mestrado.

**Resultados:**

Identificou-se 79 instituições do país, com amostra final de 67 programas avaliados, os quais foram classificadas em nove tipos a partir do uso dos princípios das políticas afirmativas em mestrados acadêmicos e profissionais.

**Conclusão:**

Os cursos de pós-graduação em Enfermagem apresentam desequilíbrio quanto à implementação de políticas afirmativas em suas ofertas de vagas regulares, bem como nas questões de equidade no acesso regional e de diversidade nos grupos sociais contemplados.

## RESUMEN

**Objetivo:**

Mapear la inclusión de políticas afirmativas en los cursos de Postgrado en Enfermería de Brasil.

**Método:**

Se trata de un estudio descriptivo, de base documental, realizado con informaciones recogidas en la Plataforma Sucupira, a través de la Coordinación de Perfeccionamiento del Personal de Enseñanza Superior, vinculada al Ministerio de Educación. Después de la recolección de datos, realizada entre octubre de 2021 y marzo de 2022, cada convocatoria fue leída en su totalidad para extraer los siguientes elementos: nombre del programa; código del programa; nombre de la Institución de Educación Superior; sigla; Unidad Federativa; y número de vacantes para maestrías.

**Resultados:**

Se identificaron 79 instituciones en el país, con una muestra final de 67 programas evaluados y clasificados en nueve tipos, a partir del uso de principios de políticas afirmativas en maestrías académicas y profesionales.

**Conclusión:**

Los cursos de postgrado en Enfermería presentan un desequilibrio en la implementación de políticas afirmativas en sus ofertas regulares de vacantes, así como en las cuestiones de equidad para el acceso regional y diversidad de los grupos sociales contemplados.

## INTRODUCTION

Affirmative action (AF) public policies, such as the Quota Law, carried out by offering places in public universities in the country, have already participated in political debates since the 1990s, when the inclusion of Afro-Brazilians in social spaces was on the docket. During this period, the demand for plurality in public spaces and the discrimination of this part of the population gave rise to discussions on public policies for equal opportunities and on reorientations in the educational system^(1,2)^.

Through historical perception, it was observed that the conservative way of maintaining the privileged classes in the field of educational, social and media resources, recurrent for generations, provided the practice of discriminatory processes in multiple scenarios. Thus, it was understood that discriminatory actions were not composed of isolated factors and events, but composed structured and complex networks of specific conditions that preceded them, such as intersectionality^(3,4)^.

Based on the requirements of the National Education Plan, which lasts for ten years, and in a constitutional manner, considering art. 214 of the Federal Constitution and Law #13,005/2014, there was the promotion of goals for overcoming educational inequalities and the elaboration of policies for all educational levels, from basic to higher education, which led to a significant expansion of offers vacancies in Federal Institutions of Higher and Technological Education, in addition to enabling forms of integration and democratization of access to public education in the country for subjects conditioned by the harmful conformations already described^(5)^.

This was solidified with the enactment of Law n.º 12,711/2012, which promoted the reservation of vacancies in higher education courses at federal institutions, linked to the Ministry of Education (MEC), complemented by Law n.º 13,409/2016, which facilitated the retention of vacancies for low-income students, black, brown, indigenous and people with disabilities, who are part of the Federation Unit where the educational institution is located, according to the records of the Brazilian Institute of Geography and Statistics (IBGE)^(2,6)^.

Although the Brazilian population is made up of 47.7% whites and 50.7% blacks, a 2015 study identified that the profile of postgraduate students was composed of 70.86% whites and 27.08% black. With the observation of this inequality, in 2016, Normative Ordinance MEC #13, supported by the Statute of Racial Equality and Law #12.711, designated the expansion of the ethnic and cultural diversities of postgraduate students, from the adoption of AP, giving autonomy to each institution to adhere or not to inclusion of proposals^(7,8)^.

In reference to the area of Nursing, there are several and diverse studies on the inclusion and follow-up of quota students in undergraduate courses^(9,10)^, but postgraduate studies present a lack of information in this regard. Therefore, it is pertinent that mechanisms for distributing vacancies aimed at social integration be evidenced, in order to collaborate with the discussion on equity in postgraduate studies, especially in what is in line with the objects of the four-year evaluation report of the Coordination for the Improvement of Higher Education Personnel (CAPES).

Thus, the present study aims to map the use of affirmative action policies in postgraduate Nursing courses in Brazil.

## METHODS

### Type of Study

This is a descriptive study, based on documents, with a quantitative approach, carried out with the data obtained from CAPES, linked to MEC.

### Data Source

The data set was extracted from the Sucupira platform, a tool designed to collect information on stricto sensu postgraduate programs (master’s and doctoral degrees) nationwide. Annually, the program coordinators are responsible for updating the information on the platform, in order to feed the databases on topics related to postgraduate studies, which serve as a reference for the four-year evaluation and academic transparency. The Sucupira platform is an important tool of the National Postgraduate System (SNPG), which handles the monitoring and evaluation of said programs^(11)^.

### Data Collection and Selection Criteria

Data selection was carried out between November 2021 and March 2022 on the Sucupira platform, based on information provided by graduate programs in Nursing about academic and professional master’s courses (PM) on the platform, in the year 2021. The collection was guided by a research instrument validated by the authors, composed of the items: adhesion of the Higher Education Institution (HEI) to the affirmative action policy; number of vacancies destined to affirmative actions; and types of affirmative action implemented in graduate programs.

Thus, the data was downloaded directly from the Sucupira platform, from which a table was generated, with the following variables: name of the program; program code; HEI name; HEI acronym; Federative unit; and quantitative number of vacancies in the institution’s master’s courses.

### Data Analysis

From the collected data, the mapping, quantification and identification of registered programs in the country were carried out. Then, the information was exported to the Microsoft Excel software, being evaluated by descriptive statistics and presented in graphs and tables, considering, in the presentation of the classifications, the groups benefited by the AP, which are generally qualified, based on on characteristics such as race or ethnicity, gender, social class and disability.

The rationale for the discussion on the collected texts included articles indexed in the SciELO, LILACS and BDENF databases, using the descriptors: “public policy”; “Ethnic groups”; “graduate nursing education”; “teaching”; and “social inclusion”.

### Ethical Aspects

Because it is a descriptive study, supported by secondary data in the public domain, there was no need to file ethical questions by the Research Ethics Committee with Human Beings, however the analysis was carried out with scientific rigor, in view of the production of truthful and meaningful results.

## RESULTS

A total of 79 institutions were found that offer academic and professional master’s (PM) courses at the postgraduate level in Nursing in Brazil, considering the research outlines. However, one program was excluded for having only a doctoral level record and another 11 for not adhering to AP. Thirty-one programs were identified that did not offer doctoral vacancies, only academic and professional master’s degrees, but offered AP vacancies in their selections, while 36 programs had the same number of vacancies for PM courses and doctoral degrees, but their public notices did not define the number of vacancies offered for AP by academic degree.

In this scenario, in order to avoid non-scientific biases, the present work only considered evaluations at the PM level, so the final sample had 67 evaluated programs, allocated in the North, Northeast, Midwest, Southeast and South regions of Brazil.

**Figure 1.**
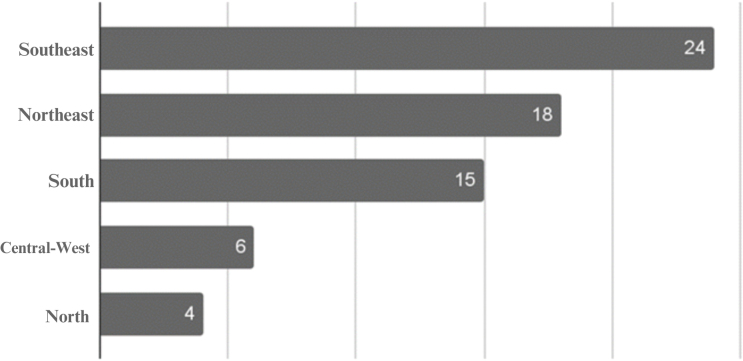
Distribution of postgraduate academic and professional master’s programs in the area of Nursing, in the regions of Brazil – Belem, PA, Brazil, 2022. Source: Sucupira Platform, (2022).

In the study, it was observed that the Southeast Region had the highest number of programs (24), considering the important factors of demographic density and the number of public universities that offer graduate programs in Nursing, followed by the Northeast, with 18 programs, South, with 15, Midwest, with six, and North, with four programs (Figure 1).

**Figure 2.**
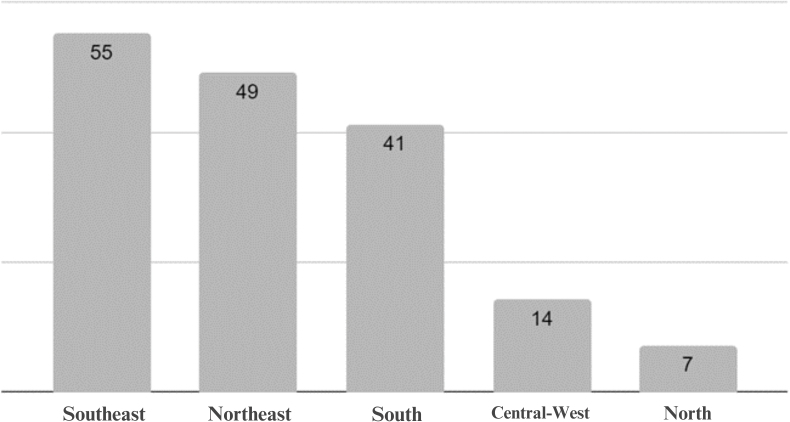
Distribution of affirmative action vacancies by postgraduate programs in the area of Nursing in Brazil - Belem, PA, Brazil, 2022. Source: Sucupira Platform, Brazil (2022).

In view of the specific vacancies for AP made available by graduate programs in Nursing in Brazil, the Southeast Region has the highest number (55 vacancies), followed by the Northeast (49), South (41), Central-West (14) and North (seven vacancies) which shows the lowest number of vacancies, as illustrated in Figure 2.

**Figure 3.**
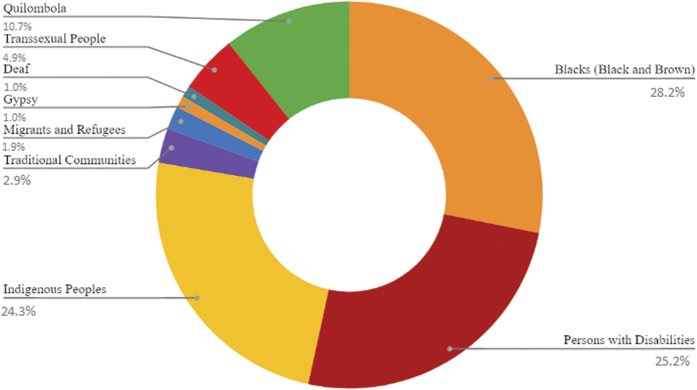
National distribution of PM courses in graduate programs in the area of Nursing, according to the type(s) of AP offered - Belem, PA, Brazil, 2022. Source: Sucupira Platform (2022).

In the same evaluation, graduate programs were separated into nine classes, considering the types of AP available in their PM courses: quilombolas; trans people; deaf; gypsies; migrants and refugees; traditional populations; indigenous; black or brown; and people with disabilities (Figure 3).

The highest distribution index observed was AP for blacks (blacks and browns), with a percentage of 28.2%, representing a quantitative of 29 programs, while the second highest percentage found was for people with disabilities (25.2%), as 26 programs adhere to this type of AP, followed by 24.3% of AF for indigenous people, arranged in 25 programs, and 10.7% of AP for quilombolas, present in 11 programs (Figure 3).

The lowest rates of offers of AP vacancies were observed for trans people, with 4.9%, distributed in five programs, for representatives of traditional populations (2.9% — three programs), for migrants and refugees (1.9% — two programs) and for deaf and gypsies (1% — one program each) (Figure 3).

Considering the regional distribution of the analyzed programs, it was verified that, among the seven states that make up the North Region, only Amazonas and Pará provide seven vacancies for AP in postgraduate courses in Nursing, exclusively for blacks (blacks and browns), for indigenous people and for people with disabilities (PwD) (Table 1).

**Table 1.** Characterization of MAP programs in the Nursing area evaluated in the five regions of Brazil, in the year 2022 – Belém, PA, Brazil, 2022.

Region	State	#Postgraduate courses	Vacancies for AP	AP types
North	Amazonas	2	6	Blacks, indigenous people and PwD
	Para	2	1	PwD
Northeast	Maranhao	1	–	–
	Piaui	1	6	Blacks, indigenous people and PwD
	Bahia	4	14	Blacks, indigenous people, quilombolas, PwD and transgender people
	Ceara	4	Not specified	Blacks, indigenous people and PwD
	Rio Grande do Norte	2	2	Blacks, indigenous people, quilombolas and PwD
	Paraiba	2	7	Blacks, indigenous people, PwD and traditional peoples and communities
	Pernambuco	2	6	Blacks, quilombolas, gypsies, indigenous people, trans people and PwD
	Alagoas	1	8	Blacks, indigenous people and PwD
	Sergipe	1	6	Blacks, indigenous people and PwD
Middle-west	Mato Grosso	1	–	–
	Mato Grosso do Sul	1	Not specified	Blacks, indigenous people and PwD
	Goiás	2	4	Black and Indigenous
	Distrito Federal	2	10	Blacks, indigenous people, quilombolas and PwD
Southeast	São Paulo	13	15	Blacks, indigenous people and PwD
	Minas Gerais	5	18	Blacks, indigenous peoples, traditional peoples and communities, trans people, PwD and refugees
	Espírito Santo	1	–	–
	Rio de Janeiro	5	22	Blacks, indigenous people and PwD
South	Paraná	5	21	Blacks, indigenous peoples and quilombolas
	Santa Catarina	4	14	Blacks, indigenous peoples, quilombolas and transgender people
	Rio Grande do Sul	6	6	Blacks, indigenous people, quilombolas, PwD and transgender people

Source: Brazil (2022).

In the Northeast Region, made up of nine states, eight programs were found (53.3% of the total number of programs found in the region) that offer AP vacancies for blacks (blacks and browns), indigenous people and PwD; four (26.7% of the total) that end offers of AP vacancies for quilombolas and/or for traditional peoples and communities; two (13.3% of the total) that offer vacancies in AP for transgender people; and one (6.7% of the total) who joined the AP vacancies for gypsy women (Table 1).

Of the four states in the Midwest Region, three ensure offers of AP vacancies in five postgraduate programs in Nursing (50% of the total programs in the region), which are aimed at blacks (blacks and browns) and indigenous people, followed by reservations for people with disabilities (made in two states — 33.3% of programs) and for quilombolas (made in one state — 16.7% of programs). Only one of the states in the region, which has a graduate program in the area, does not have vacancies for affirmative action and one of the programs does not describe the number of PA opportunities offered (Table 1).

In the Southeast, comprising four states, 24 graduate programs in Nursing were found. Of the PA vacancies available in the region, the offers cover blacks, indigenous peoples and PwD, granted in the states of São Paulo, Minas Gerais and Rio de Janeiro (in 75% of the programs), and there are vacancies for traditional peoples and communities, for trans people and for refugees in one state (Minas Gerais — 25% of programs). It is important to emphasize that, in the state of São Paulo, which has thirteen postgraduate programs in Nursing, only one provided the full number of vacancies for the PA policy, as shown in Table 1. Only one state in the region did not provided information about its entry into the affirmative action policy, in one of the public notices studied.

In the South Region, the three states offer AP vacancies in their programs, considering black, indigenous and quilombola groups (61.2% of the regional total of programs). For trans people, vacancies are offered in two states (Santa Catarina and Rio Grande do Sul — 29.9%) and, for deaf people and PwD, there is access in only one state (Rio Grande do Sul — 9%).

## DISCUSSION

The participation of students benefiting from quota policies in academic community spaces has created and revealed stigmas in the social and university environments, since there are narratives about stereotypes of inferiority of those involved, however, in fact, such AP are instruments of historical reparation, resulting from initiatives to suspend methods of social exclusion. Such a negative perception also contributes to the individual identification of the subject, producing barriers to the execution of AP in institutions and the monitoring of admissions to universities^(4,12)^.

Within the scope of postgraduate studies, APs arise from recent and unique discussions, since each program defines its guidelines and criteria for engagement, including because quota policies are flexible, which does not occur at the undergraduate level. For the most part, the programs promote the admission of candidates, based on regular stages, described in a unified public notice and ordered steps, thus the modalities of admission of wide competition and/or use of the AP are declared in each course^(13)^.

The insertion policies, which have, as an initial conception, the identification of the subjects to be admitted, define the characteristics of those who have integration needs, with the objective of aligning the premises of organization of the inclusion and exclusion practices. Such a determination places men, rich, white and heterosexual individuals as habitual and normal and defines black, poor, PwD, women and gay individuals as unequal and distinct, accustomed to the need for integration^(14)^. However, these actors are important to reduce access inequalities at the higher level^(10,15)^.

With this understanding, it is noted that meritocracy, as opposed to equity, points to a generational debate, based on social beliefs, which emphasize the logic of a Eurocentric educational system, demonstrating organizational differences, which strengthen characteristics that have already been deconstructed at different levels of education^(14)^. Precisely, valuing movements contrary to these characteristics is important in postgraduate studies, as it can bring many benefits to those involved and to society in general^(10,13,16)^.

Currently, there is a promising scenario in Nursing, with regard to racial diversity, which is occasionally beneficial to society^(13,16)^. Indeed, nursing has a significant representation of self-declared brown and black professionals, which, together, constitute 53.0% of the total number of workers in the area, in addition to indigenous professionals^(17)^. This data is relevant, as it demonstrates the contingent of students who can potentially seek postgraduate studies through offers of AP vacancies.

These points and the issues of integration and diversity in the academic environment translate into fundamental aspects in the production of relevant and contextualized knowledge^(17)^. Thus, the fact that a large percentage of nursing professionals identify themselves as brown or black may represent important trends in the diversification and enrichment of postgraduate programs in the area. Thus, postgraduate studies, in addition to being a step forward in the academic and professional training of these individuals, can significantly benefit from the variety of experiences and perspectives they bring^(10,13)^.

When analyzing the theme of gender identity in Nursing, it can be seen that this area has traditionally been and mostly female. This predominance can be attributed to several sociocultural factors, including the perception that care and empathy are characteristics often associated with being feminine. However, it is crucial to emphasize the importance of gender diversity in the profession, whether male^(17)^, whether it be those who identify as non-binary or other gender identities, which can also bring broader perspectives and approaches to patient care.

Furthermore, it is also essential to recognize and value the contribution of indigenous professionals of different age groups, who can help shape a more balanced workforce. Therefore, as we try to demonstrate here, encouraging gender, ethnicity and age diversity in nursing graduate programs is decisive^(17)^, since multiplicity, in all its manifestations, enriches the profession, making it more representative of the entire population and allowing for a more open and inclusive exchange of ideas^(10,13,16)^.

Considering these facts, it is noted that there is already a plurality in the national distribution of vacancies in the Nursing area, considering the heterogeneity of peoples, communities and groups in situations of social vulnerability, of which blacks, traditional communities, indigenous and PwD are the ones that stand out the most, evidenced by the greater visibility of these segments in postgraduate admission processes, which is in line with notes on representativeness, as signaled in other studies^(7,18)^.

In Nursing, the training of graduate professionals has shown great relevance in promoting fundamental debates for the construction of Science, in addition to allowing the qualification of professionals and composing a new perception of the autonomy of the nursing worker. In this way, the literature also highlights the social vulnerability in the implementation of postgraduate programs in Brazil, considering that the North and Midwest regions have the lowest concentration of courses, in contrast to the Southeast and Northeast, which have the highest concentration, with graduate studies concentrating the main producers of scientific research^(19)^.

Although there are gaps in the literature on aspects of insertion of AP in postgraduate programs in Nursing in Brazil, from the scenario described in this work, it was possible to identify that the North Region, despite being in a moment of ascension in the field of search^(19,20,21)^, has minimal rates of AP use when students enter their postgraduate courses, despite the fact that a large portion of the northern population is made up of indigenous populations and traditional communities, which benefit from the Quota Law at universities and in local degree courses^(10)^.

The data also clarified that the Midwest region, which has only four states in its composition, has the second largest territorial extension in Brazil and is the political division with the smallest population, has a small scope of use of AP in its postgraduate courses. -graduation. In turn, the Southeast Region, with four states, has the highest population concentration index in the country — with emphasis on the state of São Paulo, which has the most preponderant economic production in South America and is home to the only mega-metropolis in Brazil: the city of São Paulo —, is a predominantly urban region, with great ethnic, cultural and economic diversity and presents relevant methods of insertion in undergraduate courses, such as the Social Inclusion Program of the University of São Paulo (Inclusp), which reveals the use of measures to expand the access of people in vulnerable situations to the university^(9)^.

The South Region, characterized by having the smallest territorial extension at the national level, for having an influential economy and for being the second most populous division in the country, has three states in its composition, which offer AP vacancies in their programs. In the region, measures for the integration of vulnerable students have been discussed, since graduation, through the guarantee of permanence of these students in courses and the strong presence of interculturality in university spaces^(13,22,23,24)^.

Historically, the South and Southeast regions have shown the best performances in CAPES assessments, with the North and Northeast regions being marginalized, including in terms of the implementation of public policies, especially at the graduate level^(19,22)^. This fact was reflected in the last specific public notices, increasing the distance between the regions, in terms of qualification of postgraduate programs^(22)^. In this sense, it is necessary to consider the importance of such regions becoming protagonists in research on their peculiarities, an important point to raise their grades in national evaluation mechanisms.

It should be noted that the inclusion processes are relevant to the inclusion of ethnic and multicultural groups in postgraduate spaces, as these initiatives enrich dialogue, as well as reduce inequalities in education^(10,22,25)^. The expansion of the Quota Law in postgraduate courses in Nursing constitutes important instruments for the insertion and qualification of indigenous professionals, quilombolas, PwD and other actors studied here, improving the debate and increasing the level of research development in each region of the country, according to your specifics.

This study was limited to addressing the master’s programs in Brazil, based on the records of the Sucupira platform and the information provided by the graduate programs themselves, not covering the doctoral level, however. Although these postgraduate levels are stricto sensu, the master’s and doctoral programs differ in terms of objectives, requirements, expected results and duration, so it is opportune to evaluate the academic progress of the AP, depending on the integration and permanence of students in a state of vulnerability, in order to identify the connection between the levels and the barriers demonstrated, related to aspects of access, progression and effective technical and scientific training of students coming from the public in focus in this article, as well as with regard to the organization of programs in monitoring these students.

To mitigate these limitations in future studies, it would be useful to expand the research scope, seeking to include doctoral programs and additional sources of information, in addition to the Sucupira platform, to ensure more comprehensive and more accurate views of postgraduate studies in Brazil. Certainly, there is no attempt to generalize graduate programs since the characteristics and requirements of master’s programs may not represent the universe of graduate programs with the necessary accuracy.

As for the area of Nursing, this study makes it possible to visualize the result of some systems of entry of groups in a situation of social vulnerability in postgraduate studies — in master’s courses, particularly —, presenting subsidies for making new and better decisions, referring to the methods selection and vacancies. This fact adds value to the discussions on actions of social scope, an item observed by CAPES in the quadrennial evaluation procedures of the PM, being opportune in the constitution of reports on the current condition of the postgraduate programs analyzed here and in their future planning.

Additionally, this text points out the importance of discussing the meanings of integration and its processes, aiming, in addition to access, the qualification of programs and the alignment of the mentioned policies. Likewise, problematic situations already identified in graduation are highlighted, such as the question of the permanence of students enrolled in AP, which must be better managed, aiming at obtaining satisfactory results, which is far ahead of the theme of offer of inclusive vacancies that can be the subject of studies, regarding the permanence and continuity of the qualifications of these individuals.

## CONCLUSIONS

When addressing the issue of affirmative action vacancies among the 67 institutions studied here, nine significant classifications were identified, demonstrating that there is a movement towards the implementation of affirmative action policies in academic and professional master’s courses in the country, proving a dynamic with the potential to reduce (or overcome, in the future) the historical limits and barriers experienced by certain minority groups. When investigating the findings on postgraduate courses in Nursing, observing them as specialized spaces to produce knowledge, aimed at health promotion and prevention, and on the constitution of essential and unique epistemological foundations for each region, it was verified the practical difficulties in the implementation of educational policies in Brazil.

In this sense, the North and Midwest regions stood out, demonstrating the absence of initiatives to aggregate ethnicities and cultures to the profile of local nursing students, based on exchanges of knowledge and experiences with blacks, browns and indigenous peoples On the other hand, the execution of these actions in the South and Southeast is significant, confirming the logic, in which regions considered peripheral repeat the colonialist movement, regarding the production of knowledge and postgraduate training, therefore the maintenance of the Eurocentric profile is also evident among graduate students, as well as the standardization, already underway, which regulates admission by quotas to undergraduate courses.

This mapping also makes it possible, in principle, to identify and discuss possible challenges and obstacles in graduate programs in Nursing, throughout the national territory, highlighting the disparities between regions, as well as the distribution and plurality of types of eligible affirmative actions, which makes it possible to review selection processes and opportunities for advancement, in terms of equitable training and the production of information representative of regional groups, especially those with the protagonism of communities and individuals from each part of Brazil.

Moreover, the demand to produce data about and with historically excluded groups and, beyond that, about multiple public policies, which cover different human spaces, contributes to the constitution of an intercultural scientific knowledge, capable of strengthening society, which can be made possible by the insertion and recognition of the role of affirmative action policies in the practice of a more equitable education.
